# Detecting Cheaters without Thinking: Testing the Automaticity of the Cheater Detection Module

**DOI:** 10.1371/journal.pone.0053827

**Published:** 2013-01-16

**Authors:** Jens Van Lier, Russell Revlin, Wim De Neys

**Affiliations:** 1 Research Foundation - Flanders (FWO), Department of Psychology, University of Leuven, Leuven, Belgium; 2 Department of Psychology, University of California Santa Barbara, Santa Barbara, California, United States of America; 3 Centre National de la Recherche Scientifique (CNRS), LaPsyDE (CNRS Unit 3521), Paris Descartes University, Paris, France; Ecole Normale Supérieure, France

## Abstract

Evolutionary psychologists have suggested that our brain is composed of evolved mechanisms. One extensively studied mechanism is the cheater detection module. This module would make people very good at detecting cheaters in a social exchange. A vast amount of research has illustrated performance facilitation on social contract selection tasks. This facilitation is attributed to the alleged automatic and isolated operation of the module (i.e., independent of general cognitive capacity). This study, using the selection task, tested the critical automaticity assumption in three experiments. Experiments 1 and 2 established that performance on social contract versions did not depend on cognitive capacity or age. Experiment 3 showed that experimentally burdening cognitive resources with a secondary task had *no* impact on performance on the social contract version. However, in all experiments, performance on a non-social contract version did depend on available cognitive capacity. Overall, findings validate the automatic and effortless nature of social exchange reasoning.

## Introduction

Half a century of reasoning and decision making research has shown that human thinking is often biased (e.g., [1]). In a wide range of tasks, most people fail to give the correct logical response. One of the most famous examples is the Wason selection task [2]. In the standard version of the task participants are presented with four cards. For example, in the standard problem, each card has a letter on one side and a number on the other side. Hence, two of the four cards show a letter and the other two show a number (e.g., A, T, 4, 7). Participants are asked to consider the following rule (of the “If P, then Q” type) introduced by the experimenter: “If there is an A on one side, then there is a 4 on the other side.” Participants are then asked whether this rule applies to the cards presented. Thus, participants have to indicate which cards necessarily need to be turned over to be sure the rule has been followed. The correct solution focuses on the logical falsification principle; consequently the logically correct answer is to choose the ‘A’ and ‘7’ cards (which corresponds to the P and not-Q cards). This means that people have to look for instances where the rule could be violated (i.e., values that might falsify the rule). In the example the only cards that might falsify the rule are the ‘A’ and ‘7’ card. However, few individuals (typically around 10%) select the correct cards in standard descriptive selection tasks [3]–[5]. Most individuals actually select the cards that match the lexical content of the rule (e.g., ‘A’ and ‘4’ cards, which corresponds to the P and Q card).

In sharp contrast, remarkable performance boosts are consistently observed in versions of the task where the conditional rule involves a social exchange (e.g., [3], [6]–[8]). In these versions, the conditional rule fits the following template: “If you accept benefit B from me, then you must satisfy my requirement R”. For example: “If you drive my car, you have to fill up afterwards.” A person can thus be considered a cheater if that individual accepted the benefit but did not satisfy the requirement (e.g., someone who drove the car but did not fill up afterwards). Thus, searching for cheaters corresponds to choosing the logically correct cards of P and Not-Q in the selection task (e.g., you only need to check the persons who either drove the car or did not fill up the car afterwards). As a side-note, researchers have also used a switched social contract where the ‘benefit accepted’ and ‘requirement not satisfied’ cards correspond to the logical categories Q and Not-P. This is however not the logically correct response. Hence, what matters is of course detecting cheaters and not performance improvement on the Wason selection task per se. However, since we are using only standard social contracts we stick to the classic labels of performance improvement. In standard ‘social contract versions’ of the selection task 65–80% of individuals select the correct combination (P, not-Q).

To explain this facilitation, Cosmides and Tooby [7] propose the existence of a cheater detection module. This module is conceived as an adaptive algorithm in the brain that, once activated, causes individuals to automatically look for cheaters. The cheater detection module is thus a domain-specific reasoning mechanism that helps people to detect cheaters and, therefore, should result in a clear performance boost for social contract versions of the selection task. The idea of this specific module, proposed by Cosmides and Tooby [7] in their Social Contract Theory (SCT), stands in shrill contrast with the classical view that states that all behavior is based on one general learning mechanism (i.e., general cognitive capacity, intelligence, rationality). Cosmides and Tooby [8] call this classical view the Standard Social Science Model (SSSM). Note that there are a number of accounts that argue against the SSSM view. However, in this paper we focus on the crucial assumption that differentiates the SCT and the SSSM, namely that the cheater detection module works independent of general cognitive resources (i.e. automaticity, see further).

For decades, many experiments have illustrated the performance improvement on the selection task when participants have to detect cheaters in a social contract (e.g., [3], [6]–[8]). Moreover, a number of studies have presented initial evidence in support for a universal cheater detection module. For example, studies found performance boosts for social contract versions in very different cultures (e.g., Shiwiar of Ecuadorian Amazonia; [9]), and cheater detection in children as young as age four [10]. In addition, fMRI studies using the selection task showed distinct brain activity when reasoning about social exchange [11], and selective impairment of reasoning about social exchange was found in patients with bilateral damage to the limbic system [12]. Furthermore, studies also showed enhanced face recognition of cheaters [13], [14]. However, in spite of this research, debates are still ongoing about the modular hypothesis for higher order brain functions such as reasoning [8], [15], [16].

The present article focuses on the key automaticity assumption of the postulated cheater detection module. In Cosmides and Tooby’s view, modules are assumed to operate independently and are distinct from general cognitive resources [17]. As Cosmides and Tooby [18] have put it:

“When activated by content from the appropriate domain, these inference engines impose special and privileged representations during the process of situation interpretation, define specialized goals for reasoning tailored to their domain, and make available specialized inferential procedures that allow certain computations to proceed *automatically* or ‘intuitively’ and with enhanced efficiency over what a more general reasoning process could achieve given the same input.” ([18], p. 66)

In other words:

“They (*modules)* make certain kinds of inferences just as easy, effortless and ‘natural’ to humans as spinning a web is to a spider or building a dam is to a beaver.” ([17], p. 330)

Note that automaticity is a complex and multilayered concept that has come to have a very-wide use. We use the concept here as it is typically used in reasoning studies. That is, it refers to an independency from general cognitive capacity (e.g., [19]–[21]). Hence, the automaticity of the cheater detection module implies that the efficiency of cheater detection should be independent of general cognitive capacity. Put differently, reasoning in social exchange situations and looking for cheaters should not burden general cognitive resources. For clarity, note that not all modules need to operate automatically (e.g., [22]).

In this paper we examine the critical claim with respect to the role of general cognitive capacity in social exchange reasoning. In Experiment 1, we presented a large group of participants with descriptive and social contract versions of the selection task and afterwards asked them to complete the Cognitive Reflection Test (CRT; [23], [24]) to assess their cognitive capacity. We expected that performance on the descriptive version would be associated with cognitive capacity as measured by the CRT. Indeed, Cosmides and Tooby do not claim that the module will be helpful for a descriptive version. Moreover, prior correlational and dual task studies already indicated that solving a descriptive version requires abundant cognitive resources (e.g., [4], [20]). Hence, higher CRT scores can be expected to be associated with better performance on descriptive versions. However, the crucial prediction is that this correlation will not be observed for the social contract versions. Indeed, if the cheater detection module is automatic and does not depend on available cognitive resources, then even the cognitively least gifted individuals should manage to solve the social contract version correctly.

Experiment 2 looks at the impact of general cognitive capacity from a developmental perspective. It is known that general cognitive capacity increases throughout adolescence ([25]). Hence, performance on the resource demanding descriptive version can be expected to improve with age. However, if the cheater detection module operates automatically, performance on the social contract version can be expected to be relatively stable across age groups.

In the critical Experiment 3 we will validate the two initial correlational experiments with an experimental approach. We introduce a dual task load procedure to limit the available cognitive resources directly. Given that reasoning about a descriptive rule is expected to draw on cognitive resources, performance should decrease under a cognitive load since fewer resources will be available for the computation of the response. However, if the cheater detection module operates automatically, performance should not decrease under cognitive load when solving the social contract version.

### Ethics Statement

All experiments in this study were conducted in accordance with the Declaration of Helsinki and approved by the local ethics committee of the University of Leuven (Ethische Commissie Faculteit Psychologie en Pedagogische Wetenschappen). Written informed consent was obtained from all participants.

## Experiment 1

## Methods

### Participants

The 117 participants were all first-year psychology students at the University of Leuven, Belgium, who received course credit for taking part in the study.

### Materials

#### Selection task

Two versions of the selection task were used. Participants were presented with either the unfamiliar descriptive problem or the unfamiliar social contract problem adapted from Cosmides [3]. The problems were translated into Dutch. Each participant was presented with one problem. The unfamiliar versions were used to rule out scenario familiarity as a possible confound (see [3], for a discussion). Note that we stick to the original material used by Cosmides [3] to avoid interpretational complications that might result from altered material. We do note that other versions of the scenarios have been used in previous studies that looked at the association between selection task performance and cognitive capacity (e.g., [26]–[29]). However, there is debate on which feature in the scenarios is crucial to activate the cheater detection module proposed by the social contract theory. Hence, potential negative findings pointing to the non-automaticity of cheater detection can always be explained away by arguing that the altered material did not efficiently activate the proposed module. By adopting the original versions introduced by Cosmides [3] herself we sidestep this issue in the present study.

**Figure 1:**
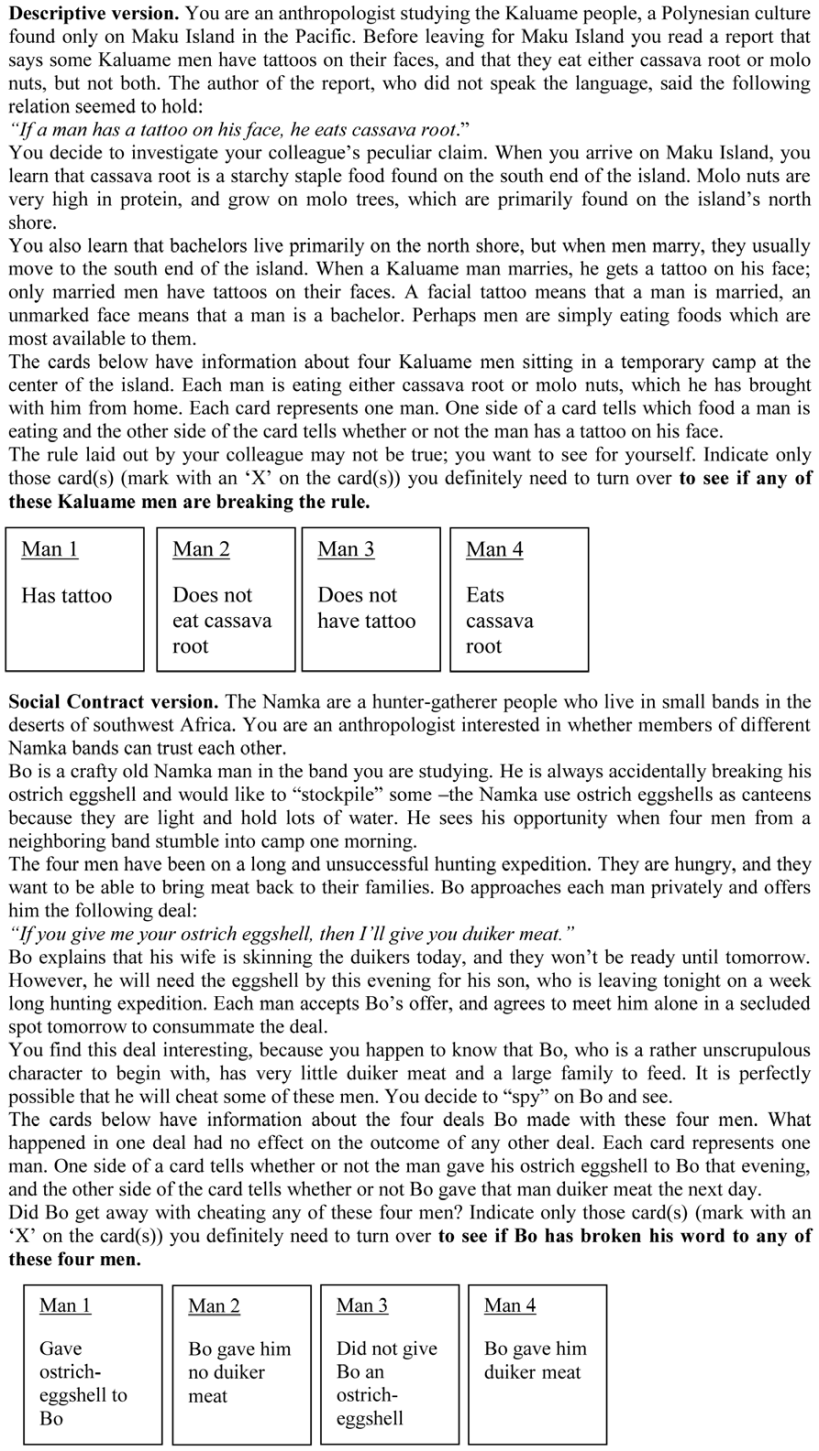
The scenario of the descriptive and social contract version.

Participants first read the following instructions: “Read the following problem carefully. Afterwards a number of “cards” will be shown to you. You will have to indicate which cards you have to turn over to check whether a certain rule has been followed or not. Mark the card(s) you think are necessary to turn over.” Then they read the scenario. The crucial difference between the two versions is that in the standard descriptive scenario version there is no social exchange involved. For the social contract version the same instructions were used. The scenario of the social contract version was of course different in the sense that it contained a social exchange (Figure 1):

#### Cognitive Reflection Task (CRT)

The CRT is a short three-item cognitive capacity test that measures the ability or disposition to resist reporting the response that first comes to mind (see [23] for details). The CRT shows good correlations with standard cognitive capacity tests (e.g., *r* = .43, with the Wonderlic Personnel Test, and *r* = .46, with the ACT; [23]). The following is an example of a CRT item:

“A bat and a ball cost $1.10 in total. The bat costs $1.00 more than the ball. How much does the ball cost? _____ cents.”

For all three items, reaching the correct solution requires the inhibition of impulsive erroneous answers. It is this suppression of the answer that easily comes to mind that heavily taxes the cognitive resources [23].

### Procedure

Participants were tested in groups of 20 to 30. Each participant had to solve one selection task problem. The selection tasks were printed on paper and were randomly distributed to the participants. Participants were requested to read the instructions carefully and to ask the experimenter when they did not understand the instructions. After completion of the selection task problem, the participants filled out the CRT.

## Results and Discussion

For the selection task, the Pollard falsification index (FI; [30]) was used as the dependent variable. The Pollard falsification index is calculated in the following way: a score of one is given for each card turned and a zero for each card not turned. The falsification index (FI) is FI = (P+not-Q) – (not-P+Q). Hence, the FI ranges between –2 and +2, with higher scores indicating better selection performance. As expected, the means of the FI of the descriptive task (*M* = 0.38, *SD* = 1.15) and social contract task (*M* = 1.17, *SD* = 0.89) differed significantly, *t*(117) = −4.150, *p*<.001, *Cohen’s d* = .77. Hence, we replicated the well-established performance boost for social contract versions.

However, the core question in this study was whether social exchange performance facilitation is independent of cognitive capacity. To assess the association between cognitive capacity as measured by the CRT and selection task performance, a correlational analysis was conducted. The score on the CRT reflects the number of items that have been correctly answered (range between 0 and 3). As expected, for the descriptive version, scores on the CRT were positively associated with the falsification index, *r* = .38, *n* = 55, *p*<.01, whereas for the social contract version, the association between CRT scores and the falsification index did not reach significance, *r* = .16, *n* = 54, *p* = .25. Average CRT scores of participants in the social contract and descriptive group did not differ, *t*(107) = .09, *p* = .93.

## Experiment 2

## Methods

### Participants

Participants were recruited from a secondary school in Belgium on a voluntary basis. A total of 191 students participated in the study. We recruited participants from two age groups: middle (grade 9/10; *n* = 98; mean age 14,8 years), and late adolescents (grade 11/12; *n* = 93; mean age 16,8 years).

### Materials

The same descriptive and social contract selection tasks as in the first experiment were used.

### Procedure

Participants were tested in groups of 20–30 in their classrooms. Participants received instructions and one selection problem. The problems were randomly distributed to participants. Participants were requested to read the instructions carefully and to ask the experimenter when they did not understand the instructions.

## Results and Discussion

**Figure 2:**
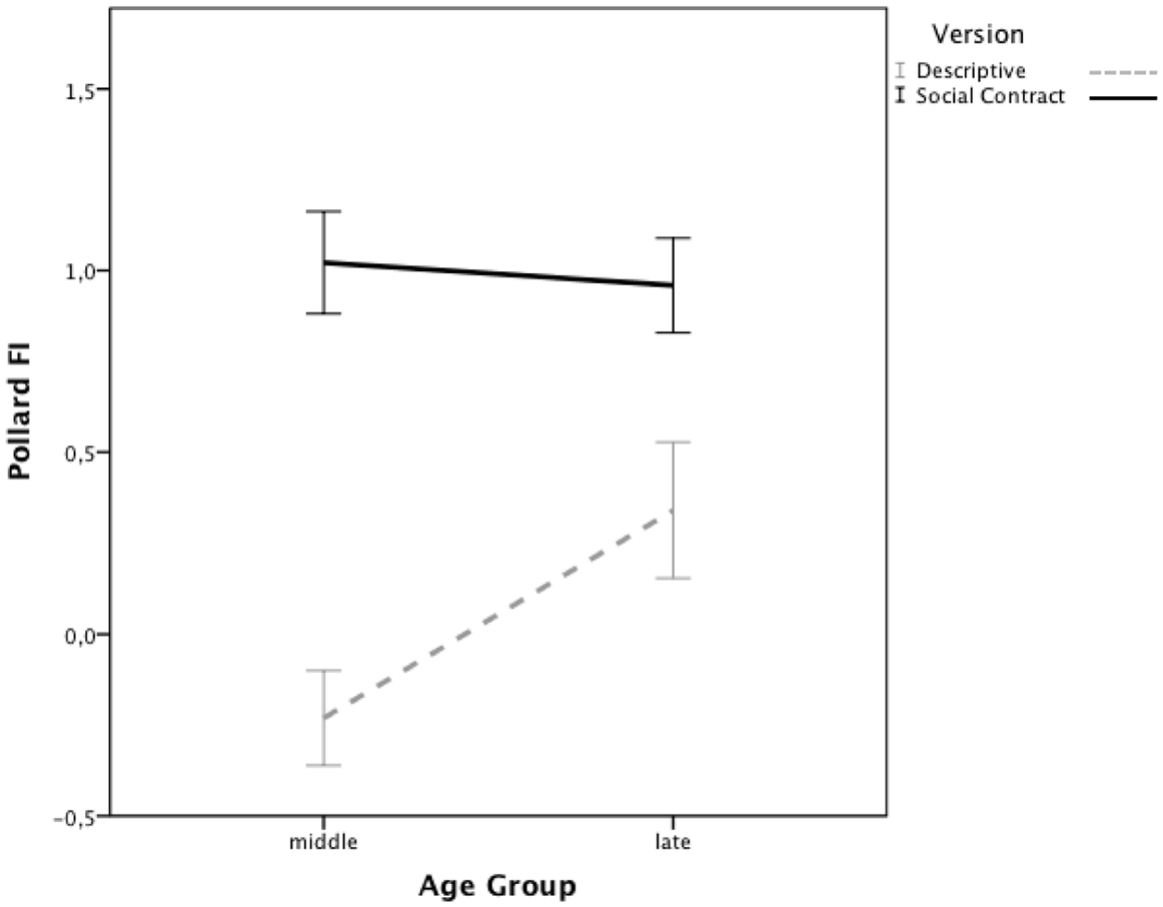
Mean falsification indices for the descriptive and social contract version across age groups. Error bars denote +/−1 standard error of the mean.

The falsification index was used as the dependent variable. To assess the effect of age group on selection task performance, the FI’s were subjected to a 2 (Age Group, between subjects) × 2 (Version, between subjects) ANOVA. There was a significant main effect of Version, *F*(1,186) = 40.53, *p*<.001, *η_p_^2^* = .18. As Figure 2 shows, consistent with previous observations, selection task performance was higher for the social contract version. There was no significant main effect for Age Group, *F*(1,186) = 3.00, *p* = .09. Crucially though, the analysis yielded a significant interaction effect between Age Group and Version, *F*(1,186) = 4.66, *p*<.05, *η_p_^2^* = .02. Planned contrasts indicated that for the descriptive version, performance was higher for late adolescents than middle adolescents, *t*(94) = − 2.57, *p*<.05, *Cohen’s d* = .53, whereas middle and late adolescents’ performance did not differ on the social contract version, *t*(93) = .33, *p* = .75.

## Experiment 3

We note that the results of experiment 1 and 2 are only correlational. As we noted above, previous correlational studies (albeit with different material) have reported conflicting findings (e.g., [20], [26], [29]). To validate our initial findings, Experiment 3 presents a stronger experimental test. This critical experimental manipulation will provide us with one of the most thorough tests of the automaticity hypothesis to date.

## Methods

### Participants

The participants were 281 first-year psychology students from the University of Leuven, Belgium, who participated in return for course credit.

### Materials

#### Selection tasks

Approximately half of the participants were presented the social contract version and the other half the descriptive version of the selection task that were used in Experiment 1 and 2 on a computer screen.

#### Dot memory task

The dot memory task is a classic spatial storage task (e.g., [31], [32]). Following the procedure of De Neys ([33], see also [34], [35]), a 3×3 matrix filled with four dots was briefly presented for 850 ms. Participants memorized the pattern and were asked to reproduce it afterwards. In the reproduction phase an empty matrix was presented on the screen, and participants used the mouse to indicate the location of the dots. A dot appeared when they clicked on the corresponding location. Clicking on the dot once more removed the dot. Participants pressed the space bar when they finished reproducing the pattern. Memorization of these dot patterns has been shown to efficiently tax cognitive resources (e.g., [32]).

### Procedure

**Figure 3:**
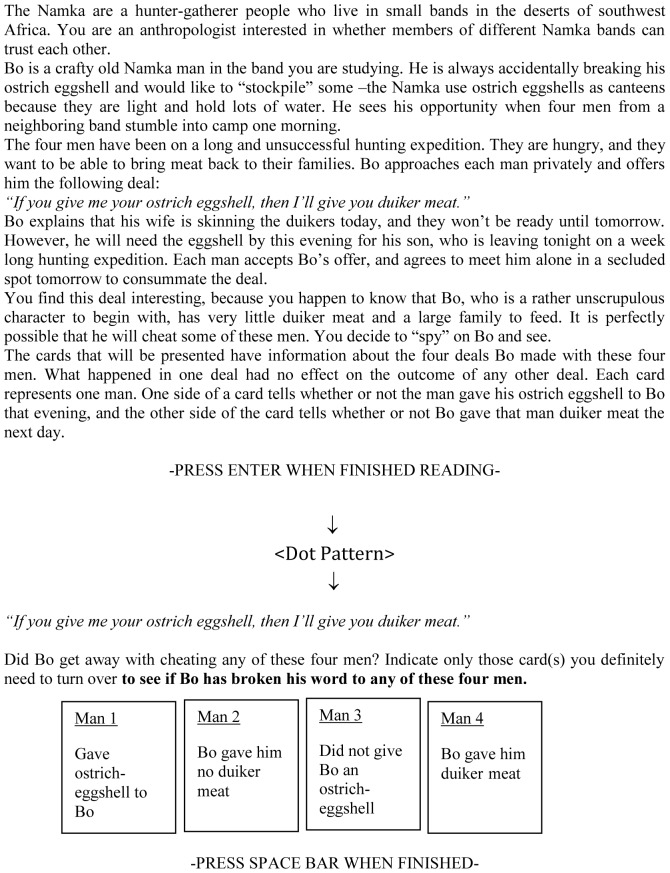
Social contract version in Experiment 3 with the sequence of the screens as presented to the participants.

Participants were tested in groups of 15 to 30 at the same time in a large computer room with an individual booth for every participant. Participants were randomly assigned to the no load (control) or load condition and to the social contract or descriptive version. After having read the general instructions that appeared on the screen participants pressed the space bar. Next, participants were familiarized with the card selection procedure. Participants used the mouse pad to indicate the cards that needed to be turned. A ‘V’ appeared under the card that had been selected. Clicking once again on the card resulted in deselecting the card. After the demonstration, the scenario of either the unfamiliar descriptive task or the unfamiliar social contract task was presented on the screen (see below for an example of the social contract version; Figure 3).

When finished reading, participants had to hit the enter key. Next, in the load group the dot pattern that participants needed to remember was presented for 850 ms. Subsequently, the highlighted rule and the cards were presented (Figure 3). Participants selected the cards as previously outlined and pressed the space bar when they finished the card selection. Finally, in the load group the empty matrix was presented and participants had to reproduce the dot pattern.

We like to stress explicitly that in the present design the load was introduced *after* participants had read the scenario. This is not a trivial issue. The mere reading and linguistic processing of the scenario will also draw on cognitive resources (e.g., [36]). Hence, ideally, the secondary task should burden resources when participants are reasoning about the card selection but it should leave the initial reading and comprehension processes unaffected. For example, note that McKinnon and Moscovitch [37] in a related study on deontic reasoning introduced a secondary task before participants had read the scenario. Therefore, the load may have affected the reading and comprehension process such that the participants were not able to represent the scenario information appropriately. To draw clear conclusions about the cognitive demands of social contract reasoning in a dual task study, it is critical that participants manage to represent the scenario properly (see [38], for a similar point). This potential confound was avoided in the present study by introducing the load after participants finished reading the scenario.

## Results and Discussion

### Dot Memory Task

Overall, the mean of correctly reproduced dots was 3.52 (*SD* = .84, range 1–4), which amounts to 88% of the dot pattern that was correctly localized. The number of dots that were correctly reproduced by participants that solved the descriptive version (*M* = 3.48, *SD* = .90) was as high as the correctly reproduced dots by participants that solved the social contract version (*M* = 3.56, *SD* = .78), *t*(133) =  −.46, *p* = .65. These results indicate that participants performed the load task properly.

### Selection Task Performance

**Figure 4:**
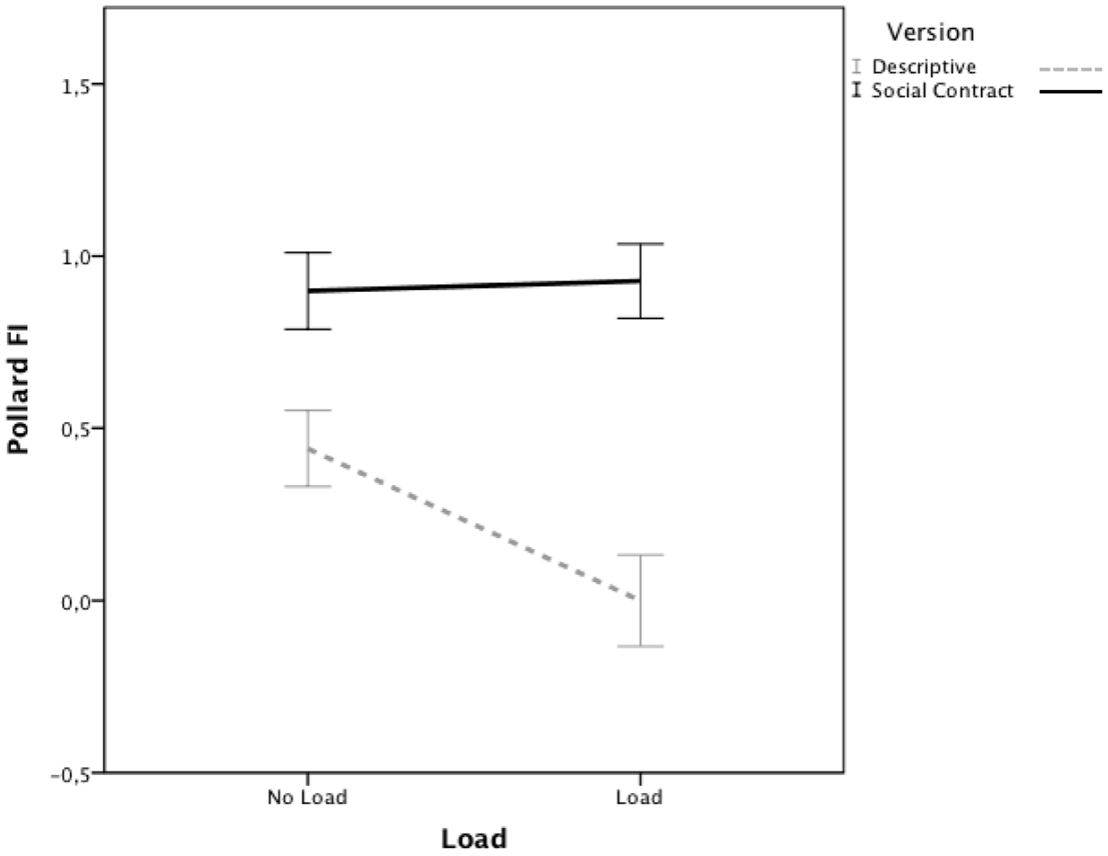
Mean falsification indices for the descriptive and social contract version in the load and no load group. Error bars denote +/−1 standard error of the mean.

The falsification index was used as the dependent variable. The FI’s were subjected to a 2 (Version, between subjects) × 2 (Load, between subjects) ANOVA. As Figure 4 shows, there was a significant main effect of Version, *F*(1,277) = 35.76, *p*<.001, *η_p_^2^* = .11, replicating again that performance on the social contract problem was better than performance on the descriptive version. There was no significant main effect of Load, *F*(1,277) = 3.18, *p* = .08. Crucially though, the interaction between Version and Load was significant, *F*(1,277) = 4.13, *p*<.05, *η_p_^2^* = .02. Consistent with previous findings, planned contrasts further established that for the descriptive version the FI decreased under load, *t*(141) = 2.58, *p*<.05, *Cohen’s d* = .43. In contrast, the FI for the social contract version was not affected by load, *t*(136) =  −.19, *p* = .85.

## General Discussion

Cosmides and Tooby have proposed the existence of a cheater detection module to explain why we are so good at detecting cheaters. This module is an adaptive algorithm in the brain that once activated causes individuals to automatically look for cheaters in social exchange. According to Cosmides and Tooby’s view of the mind, modules operate without conscious effort and are distinct from general cognitive resources [17], [18]. In the present study we tested this effortless, automatic nature of the cheater detection module.

The first experiment examined the correlation between a measure of general cognitive capacity and selection task performance. In the second experiment we looked at the cognitive capacity-performance relationship from a developmental angle, studying two groups of adolescents in a cross-sectional design. In the third experiment, available cognitive capacity was experimentally manipulated in a dual task paradigm. If the cheater detection module works automatically, we can expect that the performance facilitation on a social contract version does not depend on available cognitive capacity. By contrast, since a descriptive version should not trigger this module, we can expect an association with cognitive capacity for performance on a descriptive version. Consistent with the idea that correctly solving the descriptive version draws on cognitive capacity we observed indeed that performance decreased for people lower in cognitive capacity, for younger participants and when participants were under an experimental cognitive load. However, none of these effects were observed on the social contract version. Performance on social contract versions remained high irrespective of participants’ cognitive capacity, age or their experimental cognitive load. Taken together these findings clearly imply that solving a social contract version is automatic and does not draw on general cognitive resources. This supports the postulated automaticity of the cheater detection module.

Our findings are thus consistent with the modular view of Cosmides and Tooby. However, it will be clear that the fact that a process is automatic does not necessarily imply that it is also modular, of course (e.g., [22], [39]). Also, to the extent that other accounts make an automaticity assumption, our study does not allow us to decide between these accounts. The point is that we showed that detecting cheaters operates automatic or independent of general cognitive resources. These results falsify the SSSM view that all reasoning is based on one general mechanism. Obviously, the present study does not allow us to (and was not designed to) differentiate further between the SCT and possible alternative accounts that also share the crucial automaticity assumption.

Another point in this study is that we used the original version of the selection task that was proposed by Cosmides [3]. There is extensive debate as to whether this version of the selection task is appropriate to test the existence of a cheater detection module (e.g., [27], [28]). For example, there is evidence that individuals may solve descriptive versions, as well as deontic versions lacking the benefit-requirement structure of social contracts (e.g., [40]–[42]) and fail social contract versions [26], [28], [42]. Debate is ongoing about the original Cosmides [3] formulation of the selection task. However, it seemed necessary to test the crucial automaticity assumption of the cheater detection module with the original Cosmides [3] formulation because this presents the most basic test of their hypothesis. As we clarified, our experiments are neutral as to which specific feature of the selection task effectively activates the cheater detection module. Clearly, this also implies that the present study does not argue against the possibility that different scenarios are also facilitated while some social contract versions are not. Future research might look at this material generalization issue more closely.

We already noted that our study is related to McKinnon and Moscovitch’s [37] study on deontic reasoning. In one condition they also presented a social contract task but found that selection task performance decreased when participants were under load. Hence, they did not find support for the automaticity of cheater detection. Note that the apparent discrepancy can be readily explained. As we stated, McKinnon and Moscovitch [37] presented the load before participants had read the scenario whereas we introduced the load after participants finished reading the scenario. This is a crucial difference. Reading and comprehension of the scenario may also demand cognitive resources [36]. Hence, when given a secondary task *while* reading, it is highly likely that insufficient resources will remain to properly read the scenario. Obviously, if participants cannot properly read the scenario, the module cannot be activated. Note that to sidestep similar reading or comprehension related complications in younger age groups, we also restricted our developmental experiment to adolescents. Put bluntly: introducing the load before participants read the social contract scenario or presenting these selection tasks to very young children might amount to presenting the rule and cards *without* the scenario. Clearly, when the module is not activated, performance facilitation should not be expected (see also, [38]).

In sum, in the present paper, we have examined a crucial property of evolutionary theories of social exchange reasoning, namely the independence of a specialized cheater detection algorithm from general-purpose cognitive processes. In each of our experiments, we found that available cognitive resources were consistently related to performance on standard descriptive selection tasks, whereas cognitive resources were clearly *un*-related to performance on social contract selection tasks. Taken together, the findings lend credence to the evolutionary claim of an automatically operating cheater detection module.
